# Breast cancer risk prediction and individualised screening based on common genetic variation and breast density measurement

**DOI:** 10.1186/bcr3110

**Published:** 2012-02-07

**Authors:** Hatef Darabi, Kamila Czene, Wanting Zhao, Jianjun Liu, Per Hall, Keith Humphreys

**Affiliations:** 1 Department of Medical Epidemiology and Biostatistics, Karolinska Institutet, P.O. Box 281, Stockholm 177 71, Sweden; 2 Human Genetics, Genome Institute of Singapore, 60 Biopolis St, Singapore 138672, Singapore

## Abstract

**Introduction:**

Over the last decade several breast cancer risk alleles have been identified which has led to an increased interest in individualised risk prediction for clinical purposes.

**Methods:**

We investigate the performance of an up-to-date 18 breast cancer risk single-nucleotide polymorphisms (SNPs), together with mammographic percentage density (PD), body mass index (BMI) and clinical risk factors in predicting absolute risk of breast cancer, empirically, in a well characterised Swedish case-control study of postmenopausal women. We examined the efficiency of various prediction models at a population level for individualised screening by extending a recently proposed analytical approach for estimating number of cases captured.

**Results:**

The performance of a risk prediction model based on an initial set of seven breast cancer risk SNPs is improved by additionally including eleven more recently established breast cancer risk SNPs (*P = *4.69 × 10^-4^). Adding mammographic PD, BMI and all 18 SNPs to a Swedish Gail model improved the discriminatory accuracy (the AUC statistic) from 55% to 62%. The net reclassification improvement was used to assess improvement in classification of women into low, intermediate, and high categories of 5-year risk (*P = *8.93 × 10^-9^). For scenarios we considered, we estimated that an individualised screening strategy based on risk models incorporating clinical risk factors, mammographic density and SNPs, captures 10% more cases than a screening strategy using the same resources, based on age alone. Estimates of numbers of cases captured by screening stratified by age provide insight into how individualised screening programs might appear in practice.

**Conclusions:**

Taken together, genetic risk factors and mammographic density offer moderate improvements to clinical risk factor models for predicting breast cancer.

## Introduction

Breast cancer screening aims to detect the disease early in women and thereby reduce mortality from breast cancer. It may not be cost-effective to screen all women equally often, but rather to allocate resources disproportionately across women at different risks of developing breast cancer. To identify high- and low-risk groups, a model for estimating a woman's individual risk is needed. One of the earliest and most widely used risk models for sporadic breast cancer is the Gail model [1]. The model uses the risk factors of current age, age at menarche, age at first live birth, number of previous breast biopsies and first-degree relatives with breast cancer and converts relative risk to absolute risk through use of baseline breast cancer incidence and mortality from other causes. Several studies have assessed the contribution of adding a measure of mammographic density to breast cancer risk prediction models [2-4] because mammographic density is one of the strongest risk factors for breast cancer with a high population attributable risk [5].

Over the past decade, several common, low penetrance risk alleles for breast cancer have been identified by genome-wide association studies (GWAS), which has led to a recent increased interest in individualised risk prediction for clinical purposes [6,7]. The potential impact of adding genetic information to the Gail model has been investigated by several researchers [2,8-10]. Gail [2] added seven breast cancer risk-associated single-nucleotide polymorphisms (SNPs) to the standard Gail model and the discriminatory accuracy improved from an area under the receiver operating curve (AUC) of 60% to an AUC of 63%, which was however, less than the improvement found from adding mammographic density to the Gail model. A further 11 independent SNP associations have been recently validated in large GWAS and candidate gene studies, but their importance for risk prediction has not yet been thoroughly investigated [11-20].

Mealiffe *et al. *[8] studied prediction models based on the same seven SNPs as Gail, using data from the Women's Health Initiative clinical trial and a wider range of statistical methods. The authors studied changes in risk strata and provided evidence in favour of including genetic information in models for the prediction of breast cancer. Pharoah *et al. *[10] have further suggested that polygenic risk profiling may already provide sufficient information to justify targeting breast cancer screening to those women at highest risk. Based on a simple analytical strategy, Pashayan *et al. *[21] recently investigated the implications for individualised screening in England, using the 18 currently established breast cancer risk SNPs. The authors compared the efficiency of an individualised screening approach based on a polygenic profile, with the efficiency of a standard approach to screening, based on age alone.

We investigate the risk prediction performance of the currently established 18 breast cancer risk SNPs, empirically, in a well-characterised case-control study of breast cancer in Swedish women, with data available on mammographic density, BMI and Gail model variables. We evaluate performance of various prediction models by receiver operator characteristic curve analysis and by assessing reclassification of subjects into risk categories. We also evaluate the efficiency of individualised screening by extending the analytical strategy of Pashayan *et al. *[21] to incorporate non-genetic risk factors and to compare performance of screening programs based on equal resources with different risk-prediction models. Presentation of results stratified by age provides insight into how individualised screening programs might appear in practice.

## Materials and methods

### Data

The individuals/subjects included in the current study are drawn from a population-based case-control study of postmenopausal breast cancer in women born in Sweden aged 50 to 74 years at the time of enrolment, which was between 1 October 1993 and 31 March 1995. Controls were randomly selected from the Swedish register of the total population and were frequency matched to the expected age distribution of the cases. Details on data collection and subjects have been described previously [22]. From the original case-control study, consisting of 3,345 cases and 3,454 controls, breast density measurements were available for 1,780 cases and 1,701 controls. In all, 1,569 breast cancer cases and 1,730 healthy controls, from the original case-control study, were included in a genetic study. Among these women breast density measurements were available for 1,022 cases and 868 controls. We carried out our analysis on three subsets: women with complete data on Gail, percentage density (PD) and body mass index (BMI) variables; women with complete data on Gail and SNP variables; and women with complete data on Gail, PD, BMI and SNP variables.

The process of collecting mammographic density in the cases and controls included in this study has been described elsewhere [23]. In short, medio-lateral oblique views were used. For controls, the side was chosen randomly, whereas for cases the side contralateral to the tumour was used. The density resolution was set at 12-bit spatial resolution. Cumulus [24], a computer-assisted thresholding technique, was used to assess density on digitised film mammograms. For each image, a (single) trained observer set the appropriate gray-scale threshold levels defining the edge of the breast and distinguishing dense from non-dense tissue. The software calculated the total number of pixels within the entire region of interest and within the region identified as dense. The PD was then calculated from these values (dense area/total breast area). The images were measured together with approximately the same amount of images for healthy, control women and the reader was blinded to case-control status. A random 10% of the images were included as replicates to assess the intra-observer reliability, which was high with a Spearman rank correlation coefficient of 0.92.

Genotyping was performed at the National University of Singapore. Approval of the study was given by the Institutional Review Boards in Sweden and the National University of Singapore.

### Statistical analysis

Gail *et al. *[1] presented a method to estimate the probability that a woman, with a particular risk profile, in terms of age and other known risk factors will develop breast cancer during a specific time interval. The method can be used to combine case-control data with national registry data. Absolute risk is the probability that a subject who is free of the disease of interest at age a will be diagnosed with that disease in a subsequent age interval (*a,a *+ *δ*], and can be written as:

$$P\left(a,\delta ,r\left(t\right)\right)=\int_{a}^{a+\delta }h_{1}\left(t\right)r\left(t\right)\text{exp}\left\{-\int_{a}^{t}h_{1}\left(u\right)r\left(u\right)du\right\}\frac{S_{2}\left(t\right)}{S_{2}\left(a\right)}dt$$

where $S_{2}\left(t\right)=\text{exp}\left\{-\int_{0}^{t}h_{2}\left(u\right)du\right\}$ is the probability of surviving competing risks up to age *t*. In this equation the term *S_2_*(*t*)/*S_2_*(*a*) corresponds to the conditional probability of surviving other causes from age *a *to *t*, and the exponential term corresponds to surviving without breast cancer from age *a *to age *t*. At age *t*, there is an instantaneous probability *h*_1_(*t*)*r*(*t*)*dt *of developing breast cancer. The baseline hazard, *h*_1_(*t*), is estimated by multiplying age-specific breast cancer incidence rates, $h_{1}^{*}\left(t\right)$, by a conversion factor equal to one minus the population attributable risk. The age-specific hazard of dying from other causes other than breast cancer is represented by *h_2_*(*t*) and is assumed to be the same for all individuals. The population attributable risk is a function of the relative risk model *r*(*t*), and can be determined according to an approach described by Bruzzi *et al. *[25]. In the current article, similarly to as in Gail *et al. *[1], we work under the simplifying assumptions that *h*_1_, *h_2 _*and *r *are constant within five-year intervals. We estimated the age-specific breast cancer incidence rates and hazard of dying from other causes from the Swedish Cancer and Cause of Death registries, respectively [see Table A1 in Additional file 1], and treated these values as known, without error.

The Gail relative risk model [1] incorporates information on the risk factors age at menarche, age at first live birth, number of previous breast biopsies and first-degree relatives. We did have information on age at menarche and age at first live birth, but used family history (binary) and benign breast disease (binary), respectively, as proxies for number of first-degree relatives and the number of previous breast biopsies. In our risk-prediction models, effect estimates for Gail risk factors [1], PD, BMI and the genetic markers were retrieved from literature, except for the two Gail proxy variables. We estimated the effect sizes of the proxy variables using our own data by fitting a logistic model with both main effects included in a model which included an offset term of combined effect from age at menarche and age of first live birth, based on published effect estimates. We assumed a multiplicative penetrance model for the breast cancer-associated SNPs. In order to provide relative odds of 1.0 or more for disease-associated alleles, where necessary the genotype scores were recoded such that the low-risk homozygote represented the baseline [2]. For SNPs with effect estimates from multiple sources [11-20], we used the inverse variance method ([26]; pp.375) to obtain a weighted average of effect estimates from the separate studies.

Mammographic density has been consistently shown to be strongly associated with breast cancer and has previously been considered in breast cancer risk-prediction models [3-5]. Due to the strong negative correlation between body size and mammographic density, the effect of density on breast cancer risk is underestimated if body size is not adjusted for. We therefore included BMI, together with PD in our risk prediction models. We used effect sizes obtained externally from a large sample of postmenopausal women, from [27], as estimates of risk (odds ratios) of breast cancer according to percent mammographic density (six categories), adjusted for BMI and as estimates of risk of breast cancer according to BMI (five categories), adjusted for density (Table 1).

**Table 1:** Effect sizes for the 18 genomic loci, percentage mammographic density, body mass index and clinical risk factors, used for risk prediction.

dbSNP No	Chromosome	OR^a^	Reference First author (Year)	OR (95%CI)^b ^	*P* ^c^
rs11249433	1	1.12	Turnbull (2010), Thomas (2009)	1.12 (1.00 to 1.25)	4.3 × 10^-2^
rs1045485	2	1.14	Turnbull (2010), Cox (2007)	1.08 (0.90 to 1.28)	4.1 × 10^-1^
rs13387042	2	1.15	Turnbull (2010), Thomas (2009), Stacy (2007)	1.21 (1.08 to 1.34)	6.0 × 10^-4^
rs4973768	3	1.11	Turnbull (2010), Ahmed (2009)	1.04 (0.94 to 1.16)	4.2 × 10^-2^
rs10941679	5	1.19	Turnbull (2010), Stacy (2007)	1.19 (1.06 to 1.34)	4.0 × 10^-3^
rs889312	5	1.14	Turnbull (2010), Easton (2007)	1.14 (1.01 to 1.28)	3.5 × 10^-2^
rs2046210	6	1.27	Turnbull (2010), Zeng (2009)	1.14 (1.01 to 1.27)	2.7 × 10^-2^
rs13281615	8	1.10	Turnbull (2010), Easton (2007)	1.19 (1.07 to 1.33)	1.6 × 10^-3^
rs1011970	9	1.09	Turnbull (2010)	1.04 (0.90 to 1.21)	5.5 × 10^-1^
rs2981582	10	1.26	Turnbull (2010), Easton (2007)	1.28 (1.15 to 1.43)	6.0 × 10^-6^
rs2380205	10	1.11	Turnbull (2010)	1.04 (0.93 to 1.16)	4.9 × 10^-1^
rs10995190	10	1.16	Turnbull (2010)	1.12 (0.98 to 1.30)	9.9 × 10^-2^
rs704010	10	1.07	Turnbull (2010)	1.07 (0.96 to 1.19)	2.5 × 10^-1^
rs3817198	11	1.07	Turnbull (2010), Thomas (2009), Easton (2007)	1.01 (0.90 to 1.14)	8.1 × 10^-1^
rs614367	11	1.15	Turnbull (2010)	1.36 (1.18 to 1.58)	8.3 × 10^-4^
rs999737	14	1.09	Turnbull (2010), Thomas (2009)	1.09 (0.96 to 1.25)	1.9 × 10^-2^
rs3803662	16	1.20	Turnbull (2010), Thomas (2009), Easton (2007), Stacy (2007)	1.27 (1.13 to 1.43)	1.0 × 10^-4^
rs6504950	17	1.05	Turnbull (2010), Ahmed (2009)	1.11 (0.98 to 1.25)	1.6 × 10^-1^
Percentage mammographic density					
			
0		1.00	Boyd (2006)		
< 10%		1.27			
10-25		2.00			
25-50		2.98			
50-75		3.70			
≥ 75		5.86			
BMI (body mass Index)					
			
< 21.79		1.00	Boyd (2006)		
21.79-23.30		1.16			
23.30-25.02		1.13			
25.02-27.64		1.28			
≥ 27.64		1.67			
Clinical factors					
			
Age at menarche		1.10	Gail (1989)		
Age at first live birth		1.24	Gail (1989)		
Benign breast disease		1.65	Estimated from Swedish Case-Control Data		
Family history		2.07	Estimated from Swedish Case-Control Data		

a Published odds ratio (For SNPs with effect estimates from multiple sources, the inverse variance method was used to obtain a weighted average of effect estimate from the separate studies)

b Per allele odds ratio (per copy of the high-risk allele in the Swedish case-control sample).

c *P*-values for tests of association based on likelihood-ratio tests, in the current Swedish case-control study.

We used the Gail approach to estimate the 5-year and 10-year absolute risk of breast cancer based on age and various combinations of genetic and non-genetic risk factors. We evaluated various models for breast cancer risk based on subsets of women with data on (i) Gail, PD and BMI variables, (ii) Gail and SNP variables and (iii) Gail, PD, BMI and SNP variables.

We used the Hosmer-Lemeshow test to assess calibration of the prediction models based on comparing observed and expected outcomes within deciles of estimated risk. As in Mealiffe *et al. *[8], we first fitted a logistic regression model with a coefficient of one for the logit of the absolute risk to estimate a location parameter to account for the case-control design. We also evaluated Brier scores [see Additional file 2]. To assess discrimination we performed receiver operating characteristic curve analysis, calculating the AUC statistic, along with DeLong's non-parametric interval for AUC, and assessed departure from a model with no diagnostic capacity using the Mann-Whithney U test. We used the non-parametric approach of DeLong *et al. *[28] to test for differences in AUC.

To assess the ability of a new test to reclassify subjects accurately into higher or lower risk categories, we evaluated the two statistics suggested by Pencina *et al. *[29] for assessing improvement in model performance accomplished by adding new explanatory variables, the net reclassification improvement (NRI) and the integrated discrimination improvement (IDI). We also examined the predictiveness curve [30].

For the English population Pashayan *et al. *[21] have evaluated the efficiency of individualised screening strategies for breast cancer based on age and polygenic risk profiles. They evaluated the number of cases potentially detectable, along with the number of women eligible for screening (in the population of women aged 35 to 79 years) based on an individualised screening strategy of screening women aged 35 to 79 years with a 2.5% 10-year risk evaluated as a function of age and polygenic profile. Their approach involves inferring points on the predictiveness curve in the population (aged 35 to 79 years) at large. We extended the procedure (see below) to evaluate the potential impact of individualised screening in Sweden. As mammography screening is offered to women aged 40 to 75 years in Sweden, we evaluated the performance of a number of individualised screening approaches against a baseline (age only) strategy of screening all women between aged 40 to 75 years. In Sweden, with its screening strategy, the 10-year absolute risk of breast cancer reaches 2.5% by age 40 years and is thereafter above 2.5% all the way up to age 75 years (absolute risk values derived from Table A1 [see Additional file 1], using (1); data not shown). In addition to a polygenic profile, we also incorporated Gail risk factors and PD in the calculation of individualised risk scores. To calculate individualised risk scores we simulated a population of 100,000 women aged 40 to 75 years, according to the age distribution of the Swedish population. For generating non-genetic risk factors for these women we sampled from our own controls, with replacement. As our data consist of postmenopausal women, for women aged younger than 50 years we were forced to make additional simplifying assumptions. We assumed that these women have the same age conditional risk factor distribution as women aged 50 years. We evaluated different screening strategies based on estimating the proportion of the population that has an individualised risk greater than a given threshold (1.5%, 2% or 2.5%) and the proportion of cases that are expected to occur within the high-risk subgroup. Evaluating at different thresholds enabled us to find screening strategies that use equal resources (% eligible for screening) but are based on different risk-prediction models. We stratified our results in five-year age intervals to shed light on how individualised screening strategies might appear in practice. Although the analytical approach for calculating the proportion of cases captured by screening does not explicitly model the process of evolving risk scores for individual women, dependence of the distributions of the non-genetic risk factors on age is incorporated and age stratification provides valuable insights.

All statistical analyses were performed using the free statistical software R [31] and R packages ROCR and PredictABEL.

## Results

We examined several models for predicting absolute risk. We examined the effects of including the four Gail variables, with modified variable definitions, which we refer to as the Swe-Gail variables, as well as the effect of including PD and BMI. In all, 18 breast cancer susceptibility loci with common risk alleles have been examined in this study (Table 1); referred to as The18 herein. We also selected out the earlier known subset of seven markers studied by Gail [2], referred to herein as The7.

We first examined the classification abilities of models with and without PD and BMI, but including Swe-Gail risk factors age at menarche, age at first live birth, family history, benign breast disease, in 1,739 cases and 1,672 controls (Table 2). Without PD and BMI we observed an AUC of 0.569 (95% confidence interval (CI) = 0.550 to 0.588), compared with an AUC of 0.602 (95% CI = 0.584 to 0.621) with PD and BMI. The difference in AUCs was statistically significant (ΔAUC = 0.033, *P = *1.17 × 10^-7^). Based on a subset of women with complete data on Gail variables and SNPs, a statistically significant improvement in AUCs was seen when adding The7 to the Swe-Gail model. Improvement was further enhanced when the recently discovered 11 SNPs were added (ΔAUC = 0.018, *P = *4.69 × 10^-4^). Furthermore, a gain in AUCs was observed from including these 11 SNPs when the baseline model also included PD and BMI. We finally selected a subset of women with complete data on Gail variables, SNPs, PD and BMI and compared the performances of the Swe-Gail model and a model additionally including PD, BMI and The18. The latter model, referred to as the full model herein, obtained an AUC of 0.619, improving the AUC by 0.067 (*P = *3.24 × 10^-9^). In this subset, with PD and BMI only we observed on AUC of 0.541 (95% CI = 0.515 to 0.568), with The18 only we observed an AUC of 0.589 (95% CI = 0.563 to 0.614) and with PD, BMI and The18 we observed an AUC of 0.600 (95% CI = 0.575 to 0.626).

**Table 2:** Areas under the receiver operating characteristic curves for different combinations of prediction models.

				OLDmodel		NEWmodel		
OLDmodel	NEWmodel	Controls	Cases	AUC (95%CI)^a^	*P*-value AUC^b^	AUC (95%CI)^a^	*P*-value AUC^b^	*P*-value ΔAUC^c^
Swe-Gail	Swe-Gail, PD, BMI	1672	1739	0.569 (0.550 - 0.588)	3.00 × 10^-12^	0.602 (0.584 - 0.621)	3.85 × 10^-25^	1.17 × 10^-7^
Swe-Gail	Swe-Gail, The7	1527	1566	0.548 (0.527 - 0.568)	4.57 × 10^-6^	0.597 (0.577 - 0.617)	9.98 × 10^-21^	7.44 × 10^-17^
Swe-Gail	Swe-Gail, The18	1527	1566	0.548 (0.527 - 0.568)	4.57 × 10^-6^	0.615 (0.595 - 0.634)	1.96 × 10^-28^	1.54 × 10^-18^
Swe-Gail, The7	Swe-Gail, The18	1527	1566	0.597 (0.577 - 0.617)	9.98 × 10^-21^	0.615 (0.595 - 0.634)	1.96 × 10^-28^	4.69 × 10^-4^
Swe-Gail	Swe-Gail, PD, BMI	856	1017	0.552 (0526 - 0.578)	1.09 × 10^-4^	0.571 (0.545 - 0.597)	1.06 × 10^-7^	2.23 × 10^-7^
Swe-Gail	Swe-Gail, PD, BMI, The7	856	1017	0.552 (0526 - 0.578)	1.09 × 10^-4^	0.604 (0.579 - 0.630)	6.95 × 10^-15^	1.19 × 10^-7^
Swe-Gail	Swe-Gail, PD, BMI, The18	856	1017	0.552 (0526 - 0.578)	1.09 × 10^-4^	0.619 (0.594 - 0.644)	6.16 × 10^-19^	3.24 × 10^-9^
Swe-Gail,PD,BMI	Swe-Gail, PD, BMI, The7	856	1017	0.571 (0.545 - 0.597)	1.06 × 10^-7^	0.604 (0.579 - 0.630)	6.95 × 10^-15^	9.50 × 10^-9^
Swe-Gail, PD, BMI	Swe-Gail, PD, BMI, The18	856	1017	0.571 (0.545 - 0.597)	1.06 × 10^-7^	0.619 (0.594 - 0.644)	6.16 × 10^-19^	1.93 × 10^-9^
Swe-Gail, PD, BMI, The7	Swe-Gail, PD, BMI, The18	856	1017	0.604 (0.579 - 0.630)	6.95 × 10^-15^	0.619 (0.594 - 0.644)	6.16 × 10^-19^	6.18 × 10^-3^

a AUC and Confidence Interval (CI) evaluated using Delongs non-parametric estimation.

b Null hypothesis of AUC = 0.5 assessed using Mann-Whitney U test.

c Null hypothesis of ΔAUC = 0 assessed using DeLongs Test.

**Figure 1:**
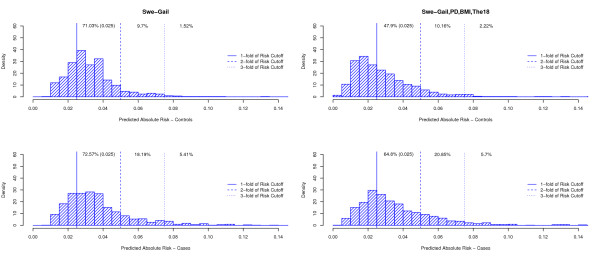
**Distributions of estimated absolute risk by case-control status using the Swe-Gail model and the full model (with displayed proportions of women with five-year absolute risks greater than (multiples of 2.5%)**.

The values of absolute five-year risk of breast cancer for the women included in our study, calculated at time of sampling/diagnosis based on the Swe-Gail model and the model additionally containing PD, BMI and The18 are plotted in Figure 1. Complementing the Gail model with PD and The18 increases the spread of the predicted absolute risks. A marked difference in distributions between cases and controls was observed for the full model. The means of the absolute five-year risks were 3.69% and 2.84% for cases and controls, respectively. Of the controls and the cases, 47.9% and 64.8%, respectively, had a five-year absolute risk higher than 2.5%. The difference in distributions between cases and controls was more subtle for the Swe-Gail model.

Assuming three risk categories, we used reclassification tables to compare pairs of models in terms of their assignment of women to low (0,ε_1_), intermediate (ε_1_,ε_2_), and elevated risk categories (ε_2_,1), based on five-year absolute risk estimates (Table 3). As cut-off values we choose ε_1 _(= 2.41%) to correspond to the first quartile of the estimated risk based on the Swe-Gail model and ε_2 _(= 4.11%) to correspond to the third quartile. The NRI value for the comparison of the Swe-Gail model with the full model was 0.170 (Z = 5.750, *P *= 8.93 × 10^-9^). In total, 46% of women were reclassified. Reclassification based on the full model was overall in the right direction, with an upward shift in risk categories for cases and a downward shift for controls. The global IDI measure was 0.004 (Z = 5.742, *P *= 9.33 × 10^-9^). Using the cut-off values suggested by Mealiffe *et al. *[8], i.e. ε_1 _= 1.5%,ε_2 _= 2%, the NRI value for comparing the same two models was estimated to be 0.193 (Z = 8.229, *P *= 2.22 × 10^-16^).

**Table 3:** Reclassification for the Swe-Gail model compared with the full model, based on cut-off values determined by first and third quartile of predicted risk by the Gail model.

Control subjects	Full model			
Swe-Gail model	Low risk (< 2.41%)	Intermediate risk (2.41%-4.11%)	High risk (> 4.11%)	Reclassified (%)
	
Low risk (< 2.41%)	170	20	10	15
Intermediate risk (2.41%-4.11%)	236	182	62	62
High risk (> 4.11%)	20	65	91	48
Cases subjects	Full model			
Swe-Gail model	Low risk (< 2.41%)	Intermediate risk (2.41%-4.11%)	High risk (> 4.11%)	Reclassified (%)
	
Low risk (< 2.41%)	155	53	17	31
Intermediate risk (2.41%-4.11%)	161	225	103	54
High risk (> 4.11%)	14	97	192	37
Total sample	Full model			
Swe-Gail model	Low risk (< 2.41%)	Intermediate risk (2.41%-4.11%)	High risk (> 4.11%)	Reclassified (%)
	
Low risk (< 2.41%)	325	72	27	24
Intermediate risk (2.41%-4.11%)	397	407	165	58
High risk (> 4.11%)	34	162	283	41

**Figure 2:**
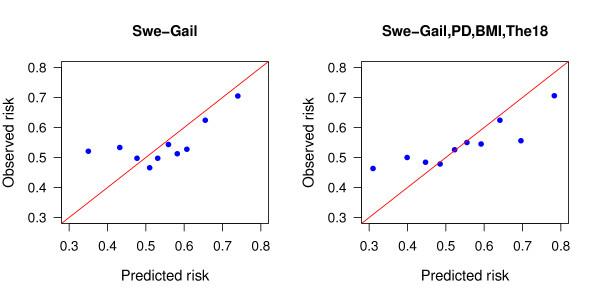
**Observed versus predicted proportions of cases for deciles of risk score for the Swe-Gail model and the full model**.

Model calibration was assessed using the Hosmer-Lemeshow approach and by calculating Brier scores. All models showed lack of fit; however, lack of model fit does not necessarily limit classification ability based on estimated risks [32]. Results for the Swe-Gail model and the full model are displayed in Tables A2 and A3 [see Additional file 1] and in Figure 2. Both the Brier score and the Hosmer-Lemeshow test statistic values indicate an improvement in goodness of fit as a result of updating the Swe-Gail model with PD, BMI and SNP data.

**Figure 3:**
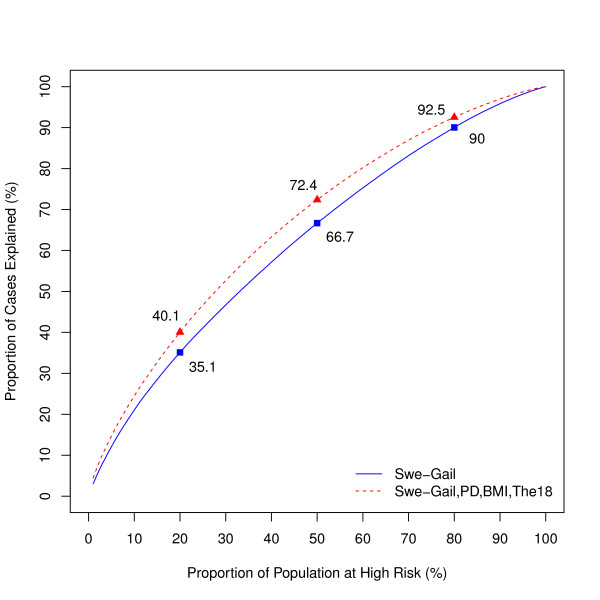
**Proportion of breast cancer cases explained by the proportion of the population at highest risk of the disease, for the Swe-Gail model and the full model**.

One way to assess the predictive power of a model is to estimate the proportions of cases that are accounted for by given percentages of the population at the highest risk [30]. Figure 3 displays these proportions based on the risk distribution generated by the Swe-Gail model, and by the full model. For the full model the proportion of cases explained by the 20% of the population at the highest risk was equal to 40.1%, compared with 35.1% for the Swe-Gail model.

For four personalised screening models, we compared the percentage of individuals eligible for screening and the percentage of cases potentially detectable by screening, for eligible cases, against the (current) approach of screening all women aged 40 to 75 years at three cut-offs of eligibility [see Table A4 of Additional file 1]. The full model with eligibility for screening defined by an absolute risk cut-off value of 2% has a slightly lower level of eligibility than the Swe-Gail model with a 2.5% cut-off for eligibility (74% and 76%, respectively), but has a substantially higher catchment; for the full model 90% of the cases are potentially screen detectable, while for the latter only 85% are potentially detectable. As a consequence of adding SNPs, BMI and PD to the risk-prediction model, resources are more efficiently re-allocated to women with high-risk profiles. With the aim of comparing screening strategies with equal resources, we also calculated the number of cases captured by screening based on the most efficient age-only screening program, and based on individualised screening using the full model when confined to including only 76% of women aged 40 to 75 years. The percentage of all cases aged 40 to 75 years covered by the age-only based program was 81%, that is 4% less than the program based on the Swe-Gail model and this was 10% less than the program based on the full risk-prediction model, for which 91% of cases were screened. Results are summarised in Table 4. Age stratified percentages of cases eligible for screening together with the percentages of cases covered by screening, for the three programs with 76% coverage, along with a selection of models presented in Table A4, are presented in Table A5 [see Additional file 1].

**Table 4:** Percentage of cases detectable by screening for the screening strategies with 76% eligibility.

Model	Cut-off^a^	Eligible^b ^(%)	Cases screened^c ^(%)	Mean (Sd)^d^
Age-Only	-	76	81	0.033 (-)
Swe-Gail	0.0250	76	85	0.034 (0.014)
Swe-Gail, PD, BMI, The18	0.0195	76	91	0.037 (0.026)

a Absolute risk cut-off defining eligibility for screening.

b Percentage of individuals eligible for screening according to the risk distribution estimated by the specified model.

c Percentage of cases potentially detectable by screening in the population undergoing screening.

d Mean and standard deviation (Sd) of predicted absolute risk values.

## Discussion

In the present study we have investigated the potential gain in combining SNP information with clinical information and mammographic percentage density for the prediction of absolute risk of developing breast cancer, in the Swedish population, utilising the Gail approach [1]. We have examined several models for predicting absolute risk, in particular examining the importance of variables in the Gail model, mammographic percentage density, the seven SNPs studied by Gail [2], and an additional 11 SNPs that have recently been confirmed to be associated with breast cancer risk. We provide evidence that the AUC of the risk-prediction model based on the initial seven breast cancer risk SNPs is improved by additionally including the 11 more recently established breast cancer risk SNPs (*P = *4.69 × 10^-4^). We further show that including mammographic PD, BMI and the 18 SNPs, in the baseline Swe-Gail model, is strongly associated with positive reclassification (NRI = 0.170, *P = *8.93 × 10^-9^).

The value of the AUC statistic, for assessing discrimination based on absolute risks calculated from the Gail model, which we observed is low compared with what has been observed in some studies carried out in the US. Rockhill *et al. *[33] observed an AUC of 0.58 based on the Nurse's Health Study and Gail [2] an AUC of 0.61 based on white women aged 50 years and over from the US National Health Interview Study. We note that for the standard Gail model, the standard deviation of the log relative risk estimated for our samples is lower than the value estimated in Gail [2] (0.34 compared with 0.36) and that generally an increase in variability in risk scores will be associated with an increased AUC value [34]. Studies of the original Gail model have reported AUC values ranging from 0.54 to 0.74, although the values at both ends of this interval have been observed in more 'extreme' populations (0.54 in a cohort of 70 year old and older US women [35] and 0.74 in a study of UK women aged 21 to 73 years from a UK family history clinic [36]).

In our study we observed improvements of 2 to 3% in AUC values as a result of adding mammographic density to risk-prediction models, which is slightly more than the 1% improvement observed by Tice *et al. *[3]. The increase is likely to be partially due to the good intra-observer reliability of the Cumulus method used for measuring percent density, compared with the BI-RADS method used by Tice *et al. *[3]; see [37]. Chen *et al. *[4] estimated an increase in excess of 4%, also using percent density. Mammograms/measurements of PD were available only on slightly fewer than 50% of the individuals in their study; statistical modelling was used to infer PD in the remaining subjects.

We examined the usefulness of the 18 markers on a population level, with respect to screening. Using published effect estimates for the 18 markers and the clinical variables we evaluated several approaches to individualised screening, against age only-based screening, in women aged 40 to 75 years (at different 10-year risk cut-offs for defining eligibility for screening). We showed for the Swedish female population that a personalised screening approach based on a risk prediction model incorporating age, Gail model variables, PD, BMI and 18 SNPs captures significantly more breast cancer cases than screening approaches using equal resources based on age and Gail model variables and on age alone. The individualised screening strategies investigated here correspond very loosely to a strategy where all women are screened at baseline (e.g. at age 40 years) and at a small number of occasions (e.g. shortly after menopause, and at age 65 years), in order to ascertain personalised risk and between these occasions women are recommended to attend screening at intervals tailored to their personalised risk. In practice, rather than reducing the total number of mammograms, as in Pashayan *et al. *[21], and in the simulation study herein, individualised screening might in the first line reallocate existing resources unequally across women, according to their risk.

It is now recognised that stratification according to genetic risk scores may improve the efficiency of screening programs [10]. With on-going genotyping efforts by among others, the Breast Cancer Association Consortium [38], it is likely that in the near future the number of established breast cancer risk SNPs will increase markedly, potentially making a polygenic approach to disease prevention a reality [11]. In the future other novel risk factors could potentially be incorporated into the Gail approach, such as steroid hormone levels, more detailed reproductive history and novel measures of mammographic density, such as texture features [39].

The strengths of the present study are the population-based setting with a high participation rate and the detailed information on key breast cancer risk factors, including mammographic density. To our knowledge this is the first study to assess the prediction performance of the currently established 18 breast cancer risk SNPs empirically.

There are limitations to the present study. Two of the variables in our prediction models varied slightly in definition from those used in the standard Gail model. For these variables we were forced to use internal effect estimates. Any bias in estimating discriminatory accuracy is, however, expected to be negligible. Further limitations are that the study is focused on postmenopausal women and that the family history variable used in our study is very crude. More sophisticated approaches have been described that more specifically describe the nature of the family history [40,41], using for example such variables as number and types of relatives affected with breast cancer (plus the ages at which they developed breast cancer), special risk factors such as BRCA1 and BRCA2 gene mutations and family history of cancers at other sites.

The approach used for assessing efficiency of individualised screening programs is simplistic. It assumes that women being screened are under constant surveillance and that cancer is instantaneously detectable without error. Moreover, our approach was based on further simplifying assumptions, for example that effect sizes are age independent. Related to this particular condition is our assumption that women aged less than 50 years have the same risk distribution of those women aged 50 years. However, in reality the relative risk associated with family history is higher at younger ages [42]. One way to relax our assumption would be to incorporate interaction effects between age and family history. Effects of other risk factors (e.g. breast density) may also vary with age, but the approach becomes unwieldy/estimates become unstable if we account for age-dependent effects of several risk factors. An approach to examining sensitivity of our results to our assumption, which addresses the issue more generally, is to investigate what happens when we increase/decrease the variance of the risk scores in the women aged less than 50 years by a fixed factor. When we increased the variance of the log relative risks by a fixed factor (10% increase) we observed an increase in the percentage of cases screened (approximately 1%) along with a very small increase (less than 1%) in the percentage of individuals eligible for screening, across all three considered prediction models, and advantages of the full model, compared with sub-models, were still observed (data not shown).

More refined approaches for evaluating screening strategies need to be developed and applied. It is important to incorporate breast cancer mortality as well as incidence and to at least partially reflect that breast cancer is a complex disease with a number of subtypes (which receive different treatments) and that patient survival outlooks vary. Accurately predicting an individuals risk of developing and dying from breast cancer remains a challenge. Microsimulation may prove a useful tool for accounting for the complicated processes of disease progression and detection when evaluating the efficiency of screening strategies [43]. Using microsimulation, it would be possible to assess refined strategies, for example, where screening intervals are defined as functions of breast cancer risk and to consider other aspects such as possible over-diagnosis and screening sensitivity.

## Conclusions

Taken together, genetic risk factors and mammographic density offer moderate improvements to clinical risk factor models for predicting breast cancer.

## Abbreviations

AUC: area under the receiver operating characteristic curve; BMI: body mass index; CI: confidence interval; GWAS: genome-wide association studies; IDI: integrated discrimination improvement; NRI: net reclassification improvement; PD: percentage density; SNPs: single nucleotide polymorphisms.

## Competing interests

The authors declare that they have no competing interests.

## Authors' contributions

HD, KH, KC, JL and PH were involved in planning the study. WZ and JL administrated the genotyping analysis. HD and KH performed the statistical analysis. HD, KH, KC and PH drafted the manuscript, and all the authors approved the manuscript.

## Supplementary Material

Additional file 1 **Supplementary tables. (a1) **Age-specific composite ($h_{1}^{*}\left(t\right)$) and competing mortality rates (*h_2_*(*t*)), for breast cancer using 2005 data from the Swedish cancer registry and cause of death registry (per 100,000). **(a2) **Measures of model calibration and discrimination for the Swe-Gail model and the full model. **(a3) **Expected and observed counts of case patients for subgroups of predicted risk for Swe-Gail model and the full model. **(a4) **Percentage of individuals eligible for screening and the percentage of cases potentially detectable by screening in the population undergoing screening, across different (personalised) screening strategies based on different cut-off of 10-year absolute risk for developing breast cancer. **(a5) **Percentage of individuals eligible for screening and the percentage of cases potentially detectable by screening in the population undergoing screening, across different screening strategies based on different cut-off of 10-year absolute risk for developing breast cancer, stratified by age. Click here for file

Additional file 2 **Supplementary methods**. Full methods accompanying this manuscript. Click here for file

## References

[B1] Gail MH Brinton LA Byar DP Corle DK Green SB Schairer C Mulvihill JJ Projecting individualised probabilities of developing breast cancer for white females who are being examined annually J Natl Cancer Inst 1989 81 1879 1886 10.1093/jnci/81.24.1879 2593165

[B2] Gail MH Discriminatory accuracy from single-nucleotide polymorphisms in models to predict breast cancer risk J Natl Cancer Inst 2008 100 1037 1041 10.1093/jnci/djn180 18612136 PMC2528005

[B3] Tice JA Gummings SR Ziv E Kerlikowske K Mammographic breast density and the Gail model for breast cancer risk prediction in a screening population Breast Cancer Res Treat 2005 94 115 122 10.1007/s10549-005-5152-4 16261410

[B4] Chen J Pee D Ayyagari R Graubard B Schairer C Byrne C Benichou J Gail MH Projecting absolute invasive breast cancer risk in white women with a model that includes mammographic density J Natl Cancer Inst 2006 98 1215 1226 10.1093/jnci/djj332 16954474

[B5] Vachon CM van Gils CH Sellers TA Ghosh K Pruthi S Brandt KR Pankratz VS Mammographic density, breast cancer risk and risk prediction Breast Cancer Res 2007 9 217 10.1186/bcr1829 18190724 PMC2246184

[B6] Easton DF Eeles RA Genome-wide association studies in cancer Hum Mol Genet 2008 17 R109 115 10.1093/hmg/ddn287 18852198

[B7] Gail MH Value of adding single-nucleotide polymorphism genotypes to a breast cancer risk model J Natl Cancer Inst 2009 101 959 963 10.1093/jnci/djp130 19535781 PMC2704229

[B8] Mealiffe ME Stokowski RP Rhees BK Prentice RL Pettinger M Hinds DA Assessment of clinical validity of a breast cancer risk model combining genetic and clinical information J Natl Cancer Inst 2010 102 1618 1627 10.1093/jnci/djq388 20956782 PMC2970578

[B9] Wacholder S Hartge P Prentice R Garcia-Closas M Feigelson HS Diver WR Thun MJ Cox DG Hankinson SE Kraft P Rosner B Berg CD Brinton LA Lissowska J Sherman ME Chlebowski R Kooperberg C Jackson RD Buckman DW Hui P Pfeiffer R Jacobs KB Thomas GD Hoover RN Gail MH Chanock SJ Hunter DJ Performance of common genetic variants in breast-cancer risk models N Engl J Med 2010 362 986 993 10.1056/NEJMoa0907727 20237344 PMC2921181

[B10] Pharoah PDP Antoniou AG Easton DF Ponder BAJ Polygenes, risk prediction, and targeted prevention of breast cancer N Engl J Med 2008 358 2796 2803 10.1056/NEJMsa0708739 18579814

[B11] Turnbull C Ahmed S Morrison J Pernet D Renwick A Maranian M Seal S Ghoussaini M Hines S Healey CS Hughes D Warren-Perry M Tapper W Eccles D Evans DG Breast Cancer Susceptibility Collaboration (UK) Hooning M Schutte M van den Ouweland A Houlston R Ross G Langford C Pharoah PD Stratton MR Dunning AM Rahman N Easton DF Genome-wide association study identifies five new breast cancer susceptibility loci Nat Genet 2010 42 504 507 10.1038/ng.586 20453838 PMC3632836

[B12] Cox A Dunning AM Garcia-Closas M Balasubramanian S Reed MW Pooley KA Scollen S Baynes C Ponder BA Chanock S Lissowska J Brinton L Peplonska B Southey MC Hopper JL McCredie MR Giles GG Fletcher O Johnson N dos Santos Silva I Gibson L Bojesen SE Nordestgaard BG Axelsson CK Torres D Hamann U Justenhoven C Brauch H Chang-Claude J Kropp S A common coding variant in GASP8 is associated with breast cancer risk Nat Genet 2007 39 352 328 10.1038/ng1981 17293864

[B13] Stacey SN Manolescu A Sulem P Rafnar T Gudmundsson J Gudjonsson SA Masson G Jakobsdottir M Thorlacius S Helgason A Aben KK Strobbe LJ Albers-Akkers MT Swinkels DW Henderson BE Kolonel LN Le Marchand L Millastre E Andres R Godino J Garcia-Prats MD Polo E Tres A Mouy M Saemundsdottir J Backman VM Gudmundsson L Kristjansson K Bergthorsson JT Kostic J Common variants on chromosomes 2q35 and 16q12 confer susceptibility to estrogen receptor-positive breast cancer Nat Genet 2007 39 865 869 10.1038/ng2064 17529974

[B14] Stacey SN Manolescu A Sulem P Thorlacius S Gudjonsson SA Jonsson GF Jakobsdottir M Bergthorsson JT Gudmundsson J Aben KK Strobbe LJ Swinkels DW van Engelenburg KC Henderson BE Kolonel LN Le Marchand L Millastre E Andres R Saez B Lambea J Godino J Polo E Tres A Picelli S Rantala J Margolin S Jonsson T Sigurdsson H Jonsdottir T Hrafnkelsson J Common variants on chromosome 5p12 confer susceptibility to estrogen receptor-positive breast cancer Nat Genet 2008 40 703 706 10.1038/ng.131 18438407

[B15] Thomas G Jacobs KB Kraft P Yeager M Wacholder S Cox DG Hankinson SE Hutchinson A Wang Z Yu K Chatterjee N Garcia-Closas M Gonzalez-Bosquet J Prokunina-Olsson L Orr N Willett WC Colditz GA Ziegler RG Berg CD Buys SS McCarty CA Feigelson HS Calle EE Thun MJ Diver R Prentice R Jackson R Kooperberg C Chlebowski R Lissowska J A multistage genome-wide association study in breast cancer identifies two new risk alleles at 1p11.2 and 14q24.1 (RAD51L1) Nat Genet 2009 41 579 584 10.1038/ng.353 19330030 PMC2928646

[B16] Ahmed S Thomas G Ghoussaini M Healey CS Humphreys MK Platte R Morrison J Maranian M Pooley KA Luben R Eccles D Evans DG Fletcher O Johnson N dos Santos Silva I Peto J Stratton MR Rahman N Jacobs K Prentice R Anderson GL Rajkovic A Curb JD Ziegler RG Berg CD Buys SS McCarty CA Feigelson HS Calle EE Thun MJ Newly discovered breast cancer susceptibility loci on 3p24 and 17q23.2 Nat Genet 2009 41 585 590 10.1038/ng.354 19330027 PMC2748125

[B17] Zheng W Long J Gao YT Li C Zheng Y Xiang YB Wen W Levy S Deming SL Haines JL Gu K Fair AM Cai Q Lu W Shu XO Genome-wide association study identifies a new breast cancer susceptibility locus at 6q25.1 Nat Genet 2009 41 324 328 10.1038/ng.318 19219042 PMC2754845

[B18] Gold B Kirchhoff T Stefanov S Lautenberger J Viale A Garber J Friedman E Narod S Olshen AB Gregersen P Kosarin K Olsh A Bergeron J Ellis NA Klein RJ Clark AG Norton L Dean M Boyd J Offit K Genome-wide association study provides evidence for a breast cancer risk locus at 6q22.33 Proc Natl Acad Sci USA 2008 105 4340 4345 10.1073/pnas.0800441105 18326623 PMC2393811

[B19] Hunter DJ Kraft P Jacobs KB Cox DG Yeager M Hankinson SE Wacholder S Wang Z Welch R Hutchinson A Wang J Yu K Chatterjee N Orr N Willett WC Colditz GA Ziegler RG Berg CD Buys SS McCarty CA Feigelson HS Calle EE Thun MJ Hayes RB Tucker M Gerhard DS Fraumeni JF Jr Hoover RN Thomas G Chanock SJ A genome-wide association study identifies alleles in FGFR2 associated with risk of sporadic postmenopausal breast cancer Nat Genet 2007 39 870 874 10.1038/ng2075 17529973 PMC3493132

[B20] Easton DF Pooley KA Dunning AM Pharoah PD Thompson D Ballinger DG Struewing JP Morrison J Field H Luben R Wareham N Ahmed S Healey CS Bowman R SEARCH collaborators Meyer KB Haiman CA Kolonel LK Henderson BE Le Marchand L Brennan P Sangrajrang S Gaborieau V Odefrey F Shen CY Wu PE Wang HC Eccles D Evans DG Peto J Fletcher O Genome-wide association study identifies novel breast cancer susceptibility loci Nature 2007 447 1087 1093 10.1038/nature05887 17529967 PMC2714974

[B21] Pashayan N Duffy SW Chowdhury S Dent T Burton H Neal DE Easton DF Eeles R Pharoah P Polygenic susceptibility to prostate and breast cancer: implications for personalised screening Br J Cancer 2011 104 1656 1663 10.1038/bjc.2011.118 21468051 PMC3093360

[B22] Low YL Li Y Humphreys K Thalamuthu A Li Y Darabi H Wedrén S Bonnard C Czene K Iles MM Heikkinen T Aittomäki K Blomqvist C Nevanlinna H Hall P Liu ET Liu J Multi-variant pathway association analysis reveals the importance of genetic determinants of estrogen metabolism in breast and endometrial cancer susceptibility PLoS Genet 2010 6 e1001012 10.1371/journal.pgen.1001012 20617168 PMC2895650

[B23] Tamimi RM Eriksson L Lagiou P Czene K Ekbom A Hsieh CC Adami HO Trichopoulos D Hall P Birth weight and mammographic density among postmenopausal women in Sweden Int J Cancer 2010 126 985 991 19642103 10.1002/ijc.24786

[B24] Byng JW Boyd NF Little L Lockwood G Fishell E Jong RA Yaffe MJ Symmetry of projection in the quantitative analysis of mammographic images Eur J Cancer Prev 1996 5 319 327 10.1097/00008469-199610000-00003 8972250

[B25] Bruzzi P Green SB Byar DP Brinton LA Schairer C Estimating the population attributable risk for multiple risk factors using case-control data Am J Epidemiol 1985 122 904 914 4050778 10.1093/oxfordjournals.aje.a114174

[B26] Kirkwood BR Sterne JAC Essential Medical Statistics 2003 2 Blackwell Science, Oxford

[B27] Boyd NF Martin LJ Sun L Guo H Chiarelli A Hislop G Yaffe M Minkin S Body Size, Mammographic Density, and Breast Cancer Risk Cancer Epidemiol Biomarkers Prev 2006 15 2086 2092 10.1158/1055-9965.EPI-06-0345 17119032

[B28] DeLong ER DeLong DM Clarke-Pearson DL Comparing the area under two or more correlated receiver operating characteristic curves: A non-parametric approach Biometrics 1988 44 837 845 10.2307/2531595 3203132

[B29] Pencina MJ D'Agostino RB Sr D'Agostino RB Jr Vasan RS Evaluating the added predictive ability of a new marker: from area under the ROC curve to reclassification and beyond Stat Med 2008 27 157 172 10.1002/sim.2929 17569110

[B30] So HG Sham PG A unifying framework for evaluating the predictive power of genetic variants based on the level of heritability explained PLoS Genet 2010 6 e1001230 10.1371/journal.pgen.1001230 21151957 PMC2996330

[B31] R Project http://www.r-project.org/

[B32] Pepe MS Feng Z Huang Y Longton G Prentice R Thompson IM Zheng Y Integrating the predictiveness of a marker with its performance as a classifier Am J Epidemiol 2008 167 362 368 17982157 10.1093/aje/kwm305 PMC2939738

[B33] Rockhill B Spiegelman D Byrne C Hunter DJ Colditz GA Validation of the Gail et al. model of breast cancer risk prediction and implications for chemoprevention J Natl Cancer Inst 2001 93 358 366 10.1093/jnci/93.5.358 11238697

[B34] Pharoah PD Antoniou A Bobrow M Zimmern RL Easton DF Ponder BA Polygenic susceptibility to breast cancer and implications for prevention Nat Genet 2002 31 33 36 10.1038/ng853 11984562

[B35] Vacek PM Skelly JM Geller BM Breast cancer risk assessment in women aged 70 and older Breast Cancer Res Treat 2011 130 291 299 10.1007/s10549-011-1576-1 21604157

[B36] Amir E Evans DG Shenton A Lalloo F Moran A Boggis C Wilson M Howell A Evaluation of breast cancer risk assessment packages in the family history evaluation and screening programme J Med Genet 2003 40 807 814 10.1136/jmg.40.11.807 14627668 PMC1735317

[B37] Boyd NF Martin LJ Yaffe MJ Minkin S Mammographic density and breast cancer risk: current understanding and future prospects Breast Cancer Research 2011 13 223 10.1186/bcr2942 22114898 PMC3326547

[B38] Yang XR Chang-Claude J Goode EL Couch FJ Nevanlinna H Milne RL Gaudet M Schmidt MK Broeks A Cox A Fasching PA Hein R Spurdle AB Blows F Driver K Flesch-Janys D Heinz J Sinn P Vrieling A Heikkinen T Aittomäki K Heikkilä P Blomqvist C Lissowska J Peplonska B Chanock S Figueroa J Brinton L Hall P Czene K Associations of breast cancer risk factors with tumor subtypes: a pooled analysis from the Breast Cancer Association Consortium studies J Natl Cancer Inst 2011 103 250 263 10.1093/jnci/djq526 21191117 PMC3107570

[B39] Manduca A Carston MJ Heine JJ Scott CG Pankratz VS Brandt KR Sellers TA Vachon CM Cerhan JR Texture features from mammographic images and risk of breast cancer Cancer Epidemiol Biomarkers Prev 2009 18 837 845 10.1158/1055-9965.EPI-08-0631 19258482 PMC2674983

[B40] Antoniou AC Cunningham AP Peto J Evans DG Lalloo F Narod SA Risch HA Eyfjord JE Hopper JL Southey MC Olsson H Johannsson O Borg A Pasini B Radice P Manoukian S Eccles DM Tang N Olah E Anton-Culver H Warner E Lubinski J Gronwald J Gorski B Tryggvadottir L Syrjakoski K Kallioniemi OP Eerola H Nevanlinna H Pharoah PD The BOADICEA model of genetic susceptibility to breast and ovarian cancers: updates and extensions British J Cancer 2008 98 1457 1466 10.1038/sj.bjc.6604305 PMC2361716 18349832

[B41] Tyrer J Duffy SW Cuzick J A breast cancer prediction model incorporating familial and personal risk factors Statistics in Medicine 2004 23 1111 1130 10.1002/sim.1668 15057881

[B42] Pharoah PD Day NE Duffy S Easton DF Ponder BA Family History and the risk of Breast Cancer: A systematic review and meta-analysis Int J Cancer 1997 71 800 809 10.1002/(SICI)1097-0215(19970529)71:5<800::AID-IJC18>3.0.CO;2-B 9180149

[B43] Nickson G Watson R Kavanagh A A microsimulation model of the BreastScreen Australia program Proceedings of 18th World IMACS/MODSIM Congress; 13 to 17 2009 Cairns, Australia

