# Supplementary material for: Breast cancer risk prediction and individualised screening based on common genetic variation and breast density measurement
Source: Breast Cancer Res. 2012 Feb 7;14(1):R25. doi: 10.1186/bcr3110 (PMC3496143; doi:10.1186/bcr3110)
Supplement: Additional file 1 — Supplementary tables. (a1) Age-specific composite (h1*(t)) and competing mortality rates (h2(t)), for breast cancer using 2005 data from the Swedish cancer registry and cause of death registry (per 100,000). (a2) Measures of model calibration and discrimination for the Swe-Gail model and the full model. (a3) Expected and observed counts of case patients for subgroups of predicted risk for Swe-Gail model and the full model. (a4) Percentage of individuals eligible for screening and the percentage of cases potentially detectable by screening in the population undergoing screening, across different (personalised) screening strategies based on different cut-off of 10-year absolute risk for developing breast cancer. (a5) Percentage of individuals eligible for screening and the percentage of cases potentially detectable by screening in the population undergoing screening, across different screening strategies based on different cut-off of 10-year absolute risk for developing breast cancer, stratified by age. [file bcr3110-S1.PDF]

Table A1: Age-specific composite ($h_1^*(t)$) and competing mortality rates ($h_2(t)$), for breast cancer using 2005 data from the Swedish cancer registry and cause of death registry, (per 100000).

Age	$h_1^*(t)$	$h_2(t)$
25-29	22.28	31.93
30-34	51.32	48.79
35-39	121.06	65.91
40-44	233.30	120.98
45-49	279.90	220.17
50-54	326.39	361.11
55-59	420.51	567.43
60-64	455.87	949.41
65-69	389.64	1540.31
70-74	342.50	2795.32
75-79	406.22	5475.06
≥80	255.17	14851.81

Table A2: Measures of model calibration and discrimination for the Swe-Gail model and the full model.

Model	Swe-Gail	Swe-Gail,PD,BMI,The18
AUC (95%CI)[a]	0.552 (0526 – 0.578)	0.619 (0.594 – 0.644)
Hosmer-Lemeshow[b]		
χ^2	45.77	22.82
P value	2.625 x 10^{-7}	3.603 x 10^{-3}
Brier Score[c]		
BS	0.249	0.239
$V(Y)$	0.248	0.248
V_{min}	1.077 x 10^{-4}	8.706 x 10^{-4}
V_{excess}	1.153 x 10^{-2}	1.895 x 10^{-2}
$(\bar{y}-\bar{\pi})^2$	7.439 x 10^{-23}	6.003 x 10^{-24}
$2Cov(Y,\pi)$	1.034 x 10^{-2}	2.939 x 10^{-2}
V_{excess} / V_{min}	107	21.77

a AUC and confidence interval.
b Hosmer-Lemeshow test statistic.
c Brier Score, see Additional file 2 for definitions.

Table A3: Expected and Observed counts of case patients for subgroups of predicted risk for Swe-Gail model and the full model.

Model	HL-Statistic[a]	Risk Decile[b]	Total[c]	Mean Risk[d]	Expected[e]	Observed[f]
Swe-Gail	45.77	[0.0095,0.0180)	190	0.350	66.43	99
		[0.0180,0.0227)	195	0.432	84.27	104
		[0.0227,0.0263)	189	0.477	90.08	94
		[0.0263,0.0288)	176	0.510	89.68	82
		[0.0288,0.0317)	187	0.531	99.25	93
		[0.0317,0.0356)	210	0.559	117.40	114
		[0.0356,0.0381)	166	0.582	96.55	85
		[0.0381,0.0443)	188	0.607	114.16	99
		[0.0443,0.0578)	189	0.655	123.81	118
		[0.0578,0.1391]	183	0.740	135.36	129
Swe-Gail,PD,BMI,The18	22.82	[0.0039,0.0128)	188	0.295	55.50	69
		[0.0128,0.0164)	187	0.386	72.25	76
		[0.0164,0.0205)	187	0.442	82.71	74
		[0.0205,0.0240)	188	0.489	91.90	108
		[0.0240,0.0277)	187	0.528	98.80	108
		[0.0277,0.0324)	187	0.566	105.85	107
		[0.0324,0.0379)	188	0.605	113.74	110
		[0.0379,0.0461)	187	0.647	120.90	109
		[0.0461,0.0584)	187	0.695	130.06	119
		[0.0584,0.3241]	187	0.777	145.29	137

a Hosmer-Lemeshow (HL) goodness of fit test statistic.
b Subgroups of predicted risks for each model.
c Number of observations.
d Mean predicted risk.
e Expected number of cases.
f Observed number of cases.

Table A4: Percentage of individuals eligible for screening and the percentage of cases potentially detectable by screening in the population undergoing screening, across different (personalised) screening strategies based on different cut-off of 10-years absolute risk for developing breast cancer.

Cut-off	0.025		0.020		0.015	
	Eligible	Cases Screened	Eligible	Cases Screened	Eligible	Cases Screened
Swe-Gail	76	85	93	96	99	100
The18	69	81	85	93	96	99
Swe-Gail,The18	61	79	77	89	91	97
Swe-Gail,PD,BMI,The18	61	82	74	90	88	96

Table A5: Percentage of individuals eligible for screening and the percentage of cases potentially detectable by screening in the population undergoing screening, across different screening strategies based on different cut-off of 10-years absolute risk for developing breast cancer, stratified by age.

Model	Cut-off[a]	Age Interval	Subjects[b] (%)	Distribution of Cases[c] (%)	Eligible[d] (%)	Cases Screened[e] (%)	Mean (Sd)[f]
Swe-Gail	0.025	40-45	16	13	47	60	0.026 (0.010)
		45-50	15	14	61	73	0.031 (0.012)
		50-55	15	16	90	94	0.037 (0.015)
		55-60	15	18	97	98	0.041 (0.014)
		60-65	16	18	96	98	0.039 (0.014)
		65-70	13	12	74	83	0.034 (0.014)
		70-75	10	9	63	73	0.031 (0.011)
		40-75	100	100	76	85	0.034 (0.014)
Swe-Gail,PD,BMI,The18	0.025	40-45	16	13	47	71	0.029 (0.019)
		45-50	15	14	57	79	0.034 (0.022)
		50-55	15	17	70	87	0.041 (0.026)
		55-60	15	18	75	90	0.046 (0.033)
		60-65	16	18	70	86	0.041 (0.029)
		65-70	13	12	57	78	0.035 (0.026)
		70-75	10	8	51	73	0.031 (0.021)
		40-75	100	100	61	82	0.037 (0.026)
The18	0.020	40-45	16	12	65	77	0.025 (0.010)
		45-50	15	14	79	88	0.029 (0.011)
		50-55	15	16	92	96	0.036 (0.014)
		55-60	15	18	97	99	0.039 (0.016)
		60-65	16	19	95	98	0.038 (0.015)
		65-70	13	12	87	93	0.033 (0.013)
		70-75	10	9	83	91	0.031 (0.012)
		40-75	100	100	85	93	0.033 (0.014)
Swe-Gail,PD,BMI,The18	0.020	40-45	16	13	61	82	0.029 (0.019)
		45-50	15	14	70	87	0.034 (0.022)
		50-55	15	17	81	93	0.041 (0.026)
		55-60	15	18	86	95	0.046 (0.033)
		60-65	16	18	82	93	0.041 (0.029)
		65-70	13	12	72	88	0.035 (0.026)
		70-75	10	8	66	84	0.031 (0.021)
		40-75	100	100	74	90	0.037 (0.026)
Swe-Gail,PD,BMI,The18	0.0195	40-45	16	13	63	83	0.029 (0.019)
		45-50	15	14	72	88	0.034 (0.022)
		50-55	15	17	82	94	0.041 (0.026)
		55-60	15	18	87	96	0.046 (0.033)
		60-65	16	18	83	94	0.041 (0.029)
		65-70	13	12	73	89	0.035 (0.026)
		70-75	10	8	68	85	0.031 (0.021)
		40-75	100	100	76	91	0.037 (0.026)
Swe-Gail,PD,BMI,The18	0.015	40-45	16	13	78	91	0.029 (0.019)
		45-50	15	14	85	95	0.034 (0.022)
		50-55	15	17	91	98	0.041 (0.026)
		55-60	15	18	95	99	0.046 (0.033)
		60-65	16	18	93	98	0.041 (0.029)
		65-70	13	12	87	95	0.035 (0.026)
		70-75	10	8	84	94	0.031 (0.021)
		40-75	100	100	88	96	0.037 (0.026)
Age-Only		40-45	16	12	0	0	0.025 (-)
		45-50	15	13	51	51	0.029 (-)
		50-55	15	16	100	100	0.036 (-)
		55-60	15	18	100	100	0.041 (-)
		60-65	16	19	100	100	0.039 (-)
		65-70	13	13	100	100	0.033 (-)
		70-75	10	9	100	100	0.031(-)
		40-75	100	100	76	81	0.033 (-)

a Absolute risk cut-off defining eligibility for screening.
b Percentage of subjects in age interval (out of all women aged 40-75).
c Percentage of cases within the specific age interval (out of all ages aged 40-75),
d Percentage of individuals eligible for screening according to risk distribution estimated by the specified model.
e Percentage of cases potentially detectable by screening in the population undergoing screening.
f Mean and standard deviation (Sd) of predicted absolute risk values in per age interval.
