# Supplementary material for: Breast cancer risk prediction and individualised screening based on common genetic variation and breast density measurement
Source: Breast Cancer Res. 2012 Feb 7;14(1):R25. doi: 10.1186/bcr3110 (PMC3496143; doi:10.1186/bcr3110)
Supplement: Additional file 2 — Supplementary methods. Full methods accompanying this manuscript. [file bcr3110-S2.PDF]

**Statistical Methods**

The models were also evaluated in terms of their Brier Score ($BS$) [1]

$$BS = \frac{1}{n}\sum_{i=1}^{n}(y_i - \pi_i)^2$$

that ranges in the unit interval, where n is the total number of subjects. For a given model BS estimates the expected quadratic loss incurred by the difference of observed event status $(y_i)$ and by the model predicted event probabilities $(\pi_i)$. The smaller BS is the closer are predictions to the observed data. If the predictions are always accurate, then BS equals zero. Predictions that are always wrong produce a value of 1 for the statistic. Yates [2] provides a covariance decomposition of BS as

$$BS = V(Y) + (\bar{y} - \bar{\pi})^2 + V(\pi) - 2\,\mathrm{Cov}(\mathrm{Y}, \pi).$$

The first term of the covariance decomposition is totally dependent on the data and out of external control. The second term measure if on average the probability predictions are realistic. Conditional on the level of correlation between the outcome variable and predictions, the variance among predictions can be decomposed in to two separate parts. Decomposition consists of minimum achievable variance ($V_{min}$) and excess variance ($V_{excess}$), i.e $V(\pi) = V_{min} + V_{excess}$ . The fraction $V_{excess} / V_{min}$ represents the degree of excess variation around the mean risk for cases and controls. The minimum variance is achievable when predicted probabilities among cases and controls are constant, and if these constants are different among the two groups then perfect discrimination is achieved [2], giving an AUC of unity. Generally there is no theoretical relationship between AUC and BS, since ROC-curves and AUCs are invariant under order-preserving transformations, while BS is not. Assuming perfect calibration, a theoretical functional relationship between AUC and BS can be formalized, that shows that BS values monotonically decrease with increasing AUC values [3].


[1] Gail MH, Pfeiffer RM**: On criteria for evaluating models of absolute risk**. *Biostatistics* 2005, **6**:227-39.

[2] Yates, J. F: **External correspondence: decomposition of the mean probability score.** *Organizational Behavior and Human Performance* 1982, **30**: 132-156.

[3] Ikeda M, Ishigaki T, Yamauchi K: **Relationship between Brier score and area under the binormal ROC curve.** *Comput Methods Programs Biomed* 2002, **67**:187-94.
